# Supplementary material for: Prospects for strongly coupled atom-photon quantum nodes
Source: Sci Rep. 2019 May 24;9:7798. doi: 10.1038/s41598-019-44292-2 (PMC6534555; doi:10.1038/s41598-019-44292-2)
Supplement: Supplementary file 1 — Supplementary material for [file 41598_2019_44292_MOESM1_ESM.pdf]

Supplementary material for “Prospects for strongly coupled atom-photon quantum nodes”

N Cooper, C Briddon, E Da Ros, V Nanyil, M T Greenaway and L Hackermuller

April 3, 2019

All numerical simulation methods have an associated numerical error. The magnitude of this error can be estimated via convergence testing of the simulation results. This supplementary information gives details of the convergence testing performed on the simulations used in the main article and explains how the results of this testing were used to estimate the numerical errors on the data shown. It also contains a table giving the simulation parameters used in all of the simulations described in the main article.

1 FDTD convergence testing

Direct testing confirmed that the level of discretisation of the spatial mesh was the dominant source of inaccuracy in our simulations, with variations in output for a given fractional change in mesh spacing greatly exceeding those for the same fractional changes in the time step increment or the dimensions of the volume included in the simulation.

The memory and computation time requirements of an FDTD simulation are strongly dependent on the number of spatial mesh cells used. If the number of mesh cells used along each axis is scaled by a factor ξ, then the memory requirement scales as ξ^3 and the simulation runtime as ξ^4 [1]. This scaling placed limits on how fine a spatial mesh could reasonably be used in these simulations, and an estimation of the remaining numerical error was performed. The first step towards obtaining these estimates was to study the convergence behaviour of the simulations in detail for some representative example cases.

For each fibre-hole geometry at least one test of the convergence behaviour of the simulations was carried out, based on the response of the simulated MOTL to variations in the size of the spatial mesh used for the simulation. To do this, we define a mesh scaling factor F such that, if our original simulation was run with the spatial mesh $(\Delta x, \Delta y, \Delta z) = (a, b, c)$ then a simulation with mesh scaling factor F would use $(\Delta x, \Delta y, \Delta z) = F(a, b, c)$. We then run multiple simulations with different values of F and study the corresponding variation of the apparent MOTL. The results are shown in supplementary figure 1.

Numerical inaccuracies resulting from spatial mesh size are known to have a second order (and higher) dependence on the mesh size, resulting from the use of the central difference approximation [2, 3]. The exception to this is “staircasing” errors for curved

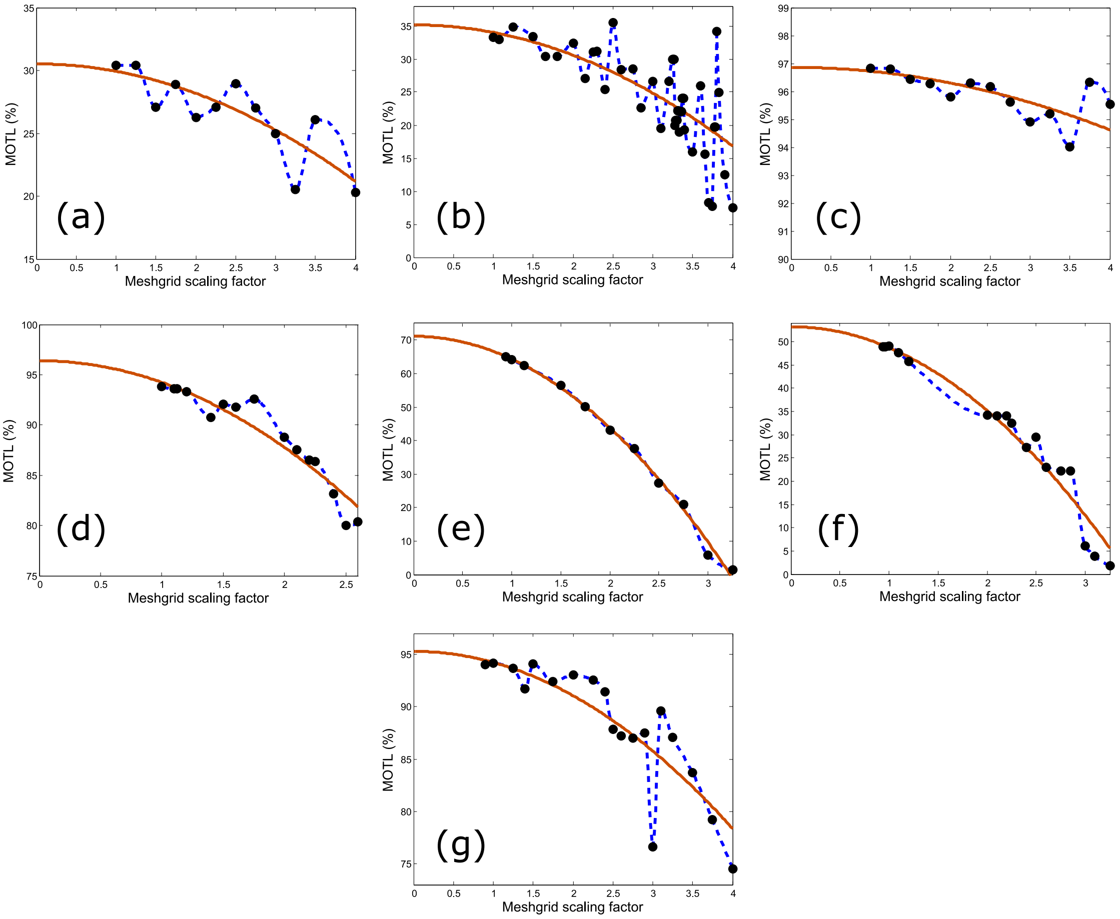

Supplementary Figure 1: Convergence testing results. The plots show simulated MOTL as a function of the mesh spacing scaling factor F, such that the mesh spacing for any given point is equal to the base mesh spacing multiplied by F. The orange line is a quadratic fit to the data, assuming errors proportional to F^2 on the individual data points. Panels (a) and (b) show results for holes with cylindrical, concave curvature (corresponding to figure 2(a)) with radii of 5 μm (a) and 7 μm (b). The base mesh spacing was $(\Delta x, \Delta y, \Delta z) = (0.04, 0.08, 0.04)$ μm for panel (a) and $(\Delta x, \Delta y, \Delta z) = (0.07, 0.07, 0.047)$ μm for panel (b). Panel (c) shows results for a rectangular hole (figure 2(b)) of length 8 μm, with a base meshgrid of $(\Delta x, \Delta y, \Delta z) = (0.08, 0.08, 0.04)$ μm. Panels (d) and (e) show results for convex, spherical surface curvature (figure 3(a)). The radius of curvature is 8 μm in (d) and 16 μm in (e). The base mesh spacing was $(\Delta x, \Delta y, \Delta z) = (0.08, 0.08, 0.058)$ μm in both panels. Panels (f) and (g) show results for holes with convex, cylindrical curvature (figure 3(b)) with a radius of 6 μm (f) and parabolic curvature (figure 4(b)) with a curvature coefficient of 0.01 μm^{-1} (g). The base mesh spacing was $(\Delta x, \Delta y, \Delta z) = (0.08, 0.08, 0.058)$ μm for panel (f) and $(\Delta x, \Delta y, \Delta z) = (0.08, 0.08, 0.08)$ μm for panel (g).

surfaces, which can cause inaccuracies with a first order dependence on mesh spacing [4]. However, in the scenarios we consider, the characteristic length scale of the physical structures involved is typically much larger than the optical wavelength, suggesting that staircasing is unlikely to be the dominant source of error. It can be seen from supplementary figure 1 that the apparent MOTL is well approximated as a quadratic function of the mesh scaling factor, in some cases with additional, oscillatory behaviour that is itself bounded by an envelope with a quadratic dependence on the mesh scaling factor. This is consistent with our expectations. We do not find evidence of a linear dependence of the apparent MOTL on mesh spacing, giving a further indication that staircasing effects do not contribute significantly to our numerical error.

2 Error estimation

Based on the results above, the assumption of quadratic scaling of the numerical error with the mesh size was used to determine the magnitude of our numerical errors. As error estimates are required for all data points, a faster method than full convergence testing was used. By determining the simulated MOTL for two different mesh spacings and extrapolating back to zero mesh spacing (assuming variation in apparent MOTL proportional to F^2), it was possible to estimate the difference between the quoted results and an extrapolated 'perfect' simulation with zero mesh spacing. This allows bounds to be placed on the deviation of our results from the true values (see supplementary figure 2). The resulting error estimate for an individual data point is then given by:

$$\text{Error} = \frac{|\text{MOTL}_1 - \text{MOTL}_2|}{F_2^2 - F_1^2} F_1^2, \tag{1}$$

where F_1 and F_2 are the scaling factors for the size of the two different spatial meshes used and MOTL_1 and MOTL_2 are the corresponding simulated MOTL values. The presence of higher order terms in the function describing the relationship between the mesh spacing and the numerical inaccuracy will on average lead to an overestimation of the error when applying equation (1), not an underestimate.

Figure 2 shows examples of the application of this method, for which we see good agreement between this method and one based on fitting all of the available convergence data. However, the applied method is more susceptible to 'noise' arising from short-scale fluctuations in apparent MOTL as a function of F, which can affect the quality of the error estimate. In order to mitigate this effect, a 3 point moving average was applied to the numerical error estimates for like points (same meshgrid and geometric form). The resulting estimates for the bounds on our numerical uncertainty are plotted as error bars in the figures in the main article.

Local fluctuations in apparent MOTL occur on a length scale that is short compared to the base mesh spacing of the simulations, with no discernible long-distance correlations. Since we always use the absolute value of ($\text{MOTL}_1 - \text{MOTL}_2$) in our error estimates, they will on average lead to an overestimate of the numerical error rather than an underestimate. As previously discussed, the same is true for the presence of higher

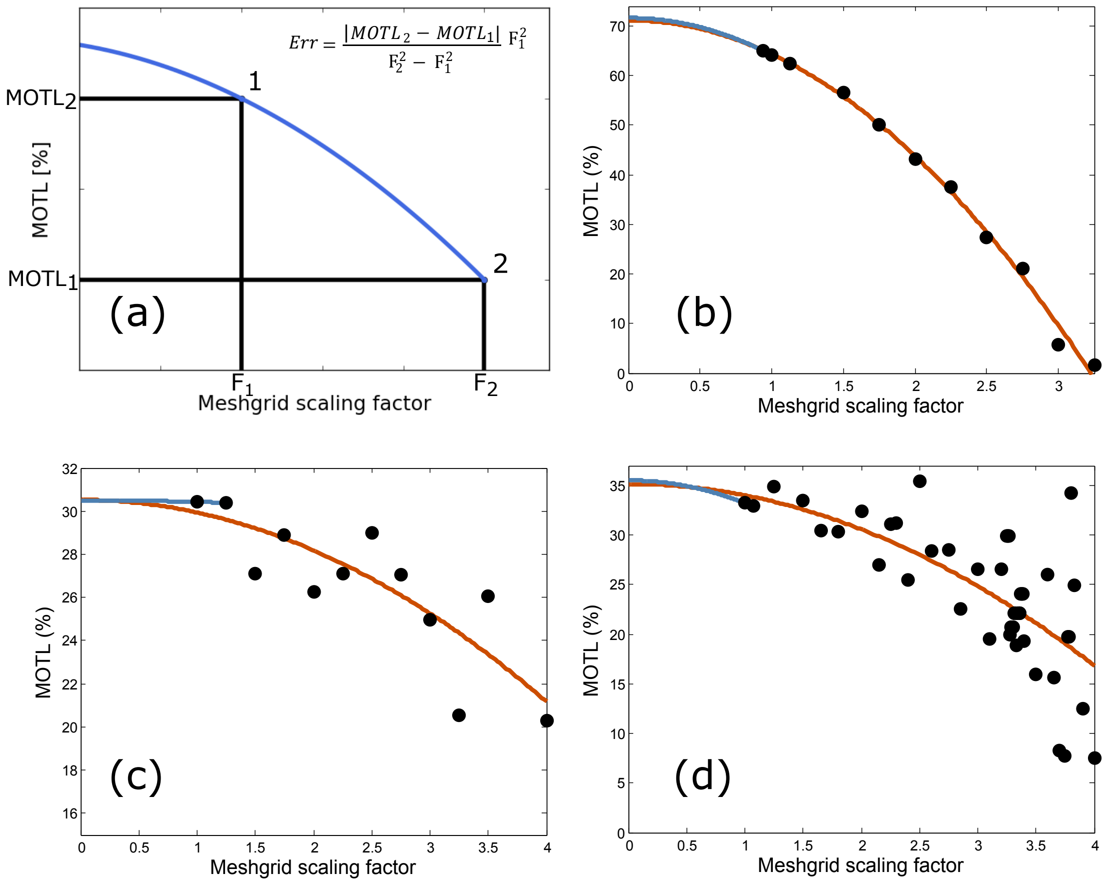

Supplementary Figure 2: (a) Sketch indicating the method of determining an error estimate from two simulations with different meshgrids. (b)—(d) Examples of implementation of this method for the cases presented in supplementary figures 1(e), 1(a) and 1(b) respectively. The blue line represents a fit to the two points with the smallest meshgrid, as used to determine the error, while the orange line represents a fit to all of the data resulting from the full convergence test. Panel (b) shows excellent agreement between the two methods, while panel (c) shows the two-point method leading to an underestimate of the error and panel (d) shows an overestimate. Since we always consider only the magnitude of the error resulting from these estimates (and neglect the sign), it can be seen that the deviations of the two-point method from the many-point fit, caused by local fluctuations in the simulated MOTL, will on average lead to an overestimation of the error rather than an underestimation. To reduce the influence of these fluctuations, we also apply a moving average to the error estimates made for points taken with the same meshgrid and sample geometry.

order terms in the scaling of the numerical inaccuracy with mesh size. Consequently the error obtained from equation (1), together with local averaging, leads to a slight overestimation of the simulation uncertainty.

3 Atom-light coupling strength in holes with convex surface curvature

Convex surface curvatures can allow high cooperativities even for larger holes. There are two reasons for this: the increased MOTL that can be achieved and a focusing effect that leads to a local enhancement of the strength of the optical field in the centre of the hole, corresponding to an increase in the value of $\phi(r)$ in equation (1) of the main article.

One example considered is a hole of length 20 μm with convex, parabolic surface curvature with a coefficient of $\frac{\delta z}{r^2} = 0.068\ \mu\text{m}^{-1}$. In this case the maximum local field strength is increased by a factor of 2.3 by the focal effects of the surfaces. The MOTL value determined for this scenario by our simulations is $(99.5^{+0.5}_{-1.3})\%$. Allowing for 8% reflection losses the corresponding values of g_1 and κ are 55 MHz and (47^{+7}_{-3}) MHz respectively. This yields $C = 4.0^{+0.2}_{-0.5}$.

Furthermore, the development of an anti-reflection coating technique for the interior hole surfaces would greatly expand the range of options available. If reflection losses are neglected, the scenario above leads to predicted values of $\kappa = 5.0$ MHz (including numerical error in MOTL up to 11.5 MHz) and $g_1 = 55$ MHz. The corresponding predicted cooperativity is C= 37 (minimum of 16 with numerical MOTL error).

Another example considered in the main article is the confinement of 260 Cs atoms within a hole of length 20 μm with convex, parabolic surface curvature with a coefficient of $\frac{\delta z}{r^2} = 0.068\ \mu\text{m}^{-1}$. This could yield collective cooperativities anywhere between $C_N = 98$ for a uniform atom distribution and $C_N = 1040$ if all of the atoms could be localised at the exact point of maximum field strength. To reach the estimate of $C_N \sim 400$ given in the main text, we assume that the atoms could be localised to a region such that the local intensity enhancement of the optical field, averaged over the atoms present, was approximately a factor of 4. This would correspond to the case of atoms confined using a dipole trap, based on light guided through the waveguide itself as discussed in the main text, whose depth was roughly 5 times the characteristic thermal energy of the trapped atomic ensemble. This is a very reasonable figure given the trap depths considered in the main text and the typical temperature of Cs atoms after magneto-optical trapping (generally of the order of 100 μK).

4 Table of simulation parameters

Supplementary table 1 shows the details of the simulation parameters used to produce all of the data displayed in the main text, including the secondary meshgrids used to estimate the numerical error.

Supplementary Table 1: Synoptic table stating all simulation parameters for the simulations presented in the main text of the paper. Figure labels including "(secondary)" refer to the additional simulations used to estimate the numerical uncertainties. The variable R represents a radius of curvature or hole radius, l the length of a rectangular hole and k the curvature coefficient for parabolic surface curvature. All distances are given in micrometers and all times in attoseconds.

**Main text figure**	**Subregion**	**Corresponding parameters** $(\Delta x, \Delta y, \Delta z, \Delta t, N_t)$
2(a)	$R = 0.25$ μm	(0.04, 0.08, 0.04, 66.7, 1680)
2(a)	1 μm $\leq R \leq 5$ μm	(0.04, 0.08, 0.04, 66.7, 1890)
2(a)	$R = 7$ μm	(0.04, 0.08, 0.04, 67.1, 2058)
2(a)	$R > 7$ μm	(0.08, 0.08, 0.0537, 99.6, 2542)
2(a) (secondary)	$R = 0.25$ μm	(0.05, 0.1, 0.05, 83.4,1360)
2(a) (secondary)	1 μm $\leq R \leq 5$ μm	(0.05, 0.1, 0.05, 82.4, 1496)
2(a) (secondary)	$R = 7$ μm	(0.05, 0.1, 0.05, 83.4, 1666)
2(a) (secondary)	$R > 7$ μm	(0.085, 0.085, 0.0571, 95.3, 2378)
2(b)	$l \leq 5$ μm	(0.08, 0.08, 0.04, 66.7, 1764)
2(b)	$l > 5$ μm	(0.08, 0.08, 0.04, 66.8, 2898)
2(b) (secondary)	$l \leq 5$ μm	(0.09, 0.09, 0.045,75.1, 1591)
2(b) (secondary)	$l > 5$ μm	(0.09, 0.09, 0.045, 75.1, 2590)
3(a)	$R < 10$ μm	(0.075, 0.075, 0.0547, 91.3, 2542)
3(a)	$R = 10$ μm	(0.08, 0.08, 0.0584, 50, 4592)
3(a)	$R > 10$ μm	(0.08, 0.08, 0.0584, 97.4, 2349)
3(a) (secondary)	$R < 10$ μm	(0.08, 0.08, 0.0584, 97.4, 2407)
3(a) (secondary)	$R \geq 10$ μm	(0.09, 0.09, 0.0657, 110, 2100)
3(b)	$R \leq 10$ μm	(0.075, 0.075, 0.0547, 91.3, 2511)
3(b)	$R > 10$ μm	(0.08, 0.08, 0.0584, 97.4, 2349)
3(b) (secondary)	$R \leq 10$ μm	(0.08, 0.08, 0.0584, 97.4, 2349)
3(b) (secondary)	$R > 10$ μm	(0.09, 0.09, 0.0657, 97.4, 2349)
4(a)	All	(0.0836, 0.0836, 0.0836, 140, 2280)
4(b)	$k = 0$	(0.08, 0.08, 0.04, 66.8, 2898)
4(b)	$0 < k \leq 0.1$ μm^{-1}	(0.08, 0.08, 0.055, 91.8, 2790)
4(b)	$k > 0.1$ μm^{-1}	(0.085, 0.085, 0.0584, 97.5, 2987)
4(b) (secondary)	$k = 0$	(0.09, 0.09, 0.045, 75.1, 2590)
4(b) (secondary)	$0 < k \leq 0.12$ μm^{-1}	(0.09, 0.09, 0.0619, 103, 2457)
4(b) (secondary)	$k > 0.12$ μm^{-1}	(0.09, 0.09, 0.0619, 103, 2808)

References

[1] A Calà Lesina, A Vaccari, P Berini, and L Ramunno. On the convergence and accuracy of the fdtd method for nanoplasmonics. *Opt. Express*, 23(8):10481–10497, Apr 2015.

[2] Optifdtd online manual. https://optiwave.com/optifdtd-manuals/fdtd-fdtd-basics/.

[3] K Warnick. An intuitive error analysis for fdtd and comparison to mom. *IEEE Antennas and Propagation Magazine*, 47:111–115, 2005.

[4] J Haggblad and O Runborg. Accuracy of staircase approximations in finite-difference methods for wave propagation. *Numerische Mathematik*, 128:741–771, 2014.
